# A Rare Presentation of Adult-Onset Bartter Syndrome: A Case Report

**DOI:** 10.7759/cureus.36120

**Published:** 2023-03-14

**Authors:** Deekshitha Alla, Meghana Krishna Kesineni, Roopeessh Vempati, Hinal Patel, Shenelle Menezes, Sai Santhosha Mrudula Alla, Devkumar Patel, Srajan Gupta, Krish Patel, Anju Pradeep

**Affiliations:** 1 Department of Medicine, Andhra Medical College, Visakhapatnam, IND; 2 Department of Internal Medicine, Gandhi Medical College & Hospital, Hyderabad, IND; 3 Department of Medicine, GMERS (Gujarat Medical Education & Research Society) Medical College, Sola, IND; 4 Department of Medicine, Spartan Health Sciences University, School of Medicine, Vieux Fort, LCA; 5 Department of Medicine, Government Medical College, Surat, IND; 6 Department of Medicine, Sri Venkateswara Medical College, Tirupati, IND; 7 Department of Medicine, Kasturba Medical College, Manipal, Manipal, IND

**Keywords:** metabolic alkalosis, hypercalciuria, hypokalemia, tubulopathy, bartter syndrome

## Abstract

Bartter syndrome is a rare, salt-wasting tubulopathy with impaired ion reabsorption in the ascending limb of the loop of Henle, which results in hypokalemia, hypochloremia, and hypercalciuria. It usually presents in neonates, with vomiting, dehydration, and failure to thrive. It results from mutations in several genes, including KCNJ1, CLCNKB, CLCNKA, BSND, and ROMK, which encode ion transporters. We report a rare presentation of adult-onset Bartter syndrome. In this case, a 27-year-old man presented to the hospital with upper and lower limb weakness. Bartter syndrome was suspected based on serum electrolytes assessment and arterial blood gas analysis. The patient was initiated on potassium chloride (KCL) infusion and potassium chloride syrup to correct hypokalemia.

## Introduction

The discovery of a novel salt-wasting tubulopathy by Bartter et al. was characterized by juxtaglomerular apparatus hyperplasia resulting in hyperreninemia, adrenal cortical hyperplasia resulting in normotensive hyperaldosteronism, and hypokalemic metabolic alkalosis as a significant event in 1962 [1].

Bartter syndrome is an autosomal recessive disorder that affects one in 1,000,000 people. Loss-of-function mutations in the ion channels of the thick ascending limb of the loop of Henle cause renal salt wasting, resulting in hypokalemia, hypochloremia, and hypercalciuria. It is classified into two types based on the phenotype, neonatal (antenatal) and classical Bartter syndrome, and five based on the genotype. Except for Types 3 and 5, all other types of Bartter syndrome are usually present in the pediatric population [2]. Clinical manifestations vary in severity and range from polyhydramnios and hypovolemia in the antenatal variant to polyuria and fatigue in the adolescent or adult-onset variant.

We present the case of a 27-year-old man, diagnosed with adult-onset Bartter syndrome. We aim to focus on the literature review and widen the gaze of physicians on this rare entity.

## Case presentation

A 27-year-old man presented to the department of general medicine with a history of weakness in his upper and lower limbs. Symptoms started two days ago and gradually increased in severity, causing difficulty rolling across the bed. During this period, he was given supportive care with no relief. He had a similar episode two months ago but had no significant family history. The patient weighed 37 kg and was 165 cm tall (BMI = 13.6 kg/m^2^). Systemic examination revealed hypotonia in all four limbs. Laboratory investigations are presented in Table 1 and Table 2. The patient had hypokalemia, metabolic alkalosis, and hypochloremia, and these features suggested the diagnosis of Bartter’s syndrome. Genetic studies were not done due to the non-availability of specialized laboratories. He was initiated on potassium chloride (KCL) infusion and potassium chloride syrup and advised to drink plenty of coconut water and consume a diet rich in potassium. Limb weakness improved greatly after volume and potassium repletion. On follow-up after 2 months, the patient’s condition improved and her electrolytes normalized with a serum sodium value of 137 mEq/L and potassium of 3.5 mEq/L. The patient was advised to eat potassium-rich foods and potassium supplements were prescribed.

**Table 1:** The results of blood analysis in the patient Na - sodium, K - potassium, Cl - chloride, Ca - calcium, Hb - hemoglobin, PCV - packed cell volume

Results of blood tests	Normal levels
Na = 140 mEq/L	135-145 mEq/L
K = 2.03 mmol/L	3.5-5 mmol/L
Cl = 84 mmol/L	96-106 mmol/L
Ca = 10.3 mmol/L	9-11.5 mmol/L
Hb = 13 gm/dL	13.2-16.6 gm/dL
PCV = 39%	38.3-48.6%

**Table 2:** The results of arterial blood gas analysis in the patient HCO3 - bicarbonate, PaCO2 - partial pressure of carbon dioxide

Parameters of Arterial Blood Gas	Normal levels
pH = 7.44	7.35-7.45
HCO_3_^- ^= 28 mEq/L	22-26 mEq/L
PaCO_2 _= 45 mmHg	35-45 mmHg

## Discussion

Bartter syndrome is an autosomal, recessive, salt-wasting disorder that is associated with a mutation in the potassium-sodium chloride cotransporter (NKCC2) or the potassium channel (ROMK) in the thick ascending limb (TAL) of the loop of Henle. The TAL's capacity to absorb sodium is reduced, which increases the distal delivery of sodium, followed by increased sodium absorption from the distal tubular segments in exchange with potassium and hydrogen ions. Volume depletion brought on by excessive salt and water loss stimulates the renin-angiotensin-aldosterone system, resulting in secondary hyperaldosteronism and hyperplasia of the juxtaglomerular apparatus. The absorption of bicarbonate ions also increases secondary to hydrogen loss, which leads to metabolic alkalosis. A positive lumen charge is eliminated by defective chloride absorption, resulting in hypercalciuria and hypermagnesuria [2].

Common biochemical abnormalities seen are hypokalemia, hypovolemia, hypochloremia, metabolic alkalosis, hypercalciuria, nephrocalcinosis, hypermagnesuria, an increased prostaglandin E2 level, and secondary hyperaldosteronism.

Bartter syndrome most typically affects children as well as adolescents. Fever, polyuria, polydipsia, vomiting, cramps, dehydration, constipation, growth delays, and failure to thrive are common clinical presentations seen in neonates. In addition to the above-mentioned symptoms, neonates also exhibit signs like sensorineural deafness, big eyes, protruding ears, strabismus, and a drooping mouth [2]. On the other hand, adults frequently present with weakness, fatigue, polyuria, polydipsia, and a salt craving [3].

Bartter syndrome is characterized by specific electrolyte and acid-base abnormalities. Hence, laboratory investigations to assess serum electrolyte levels like potassium, sodium, chloride, calcium, bicarbonate, and magnesium, are crucial for the diagnosis. Hypokalemia and hypochloremia are present in nearly all cases of Bartter syndrome. Assessment of urine electrolytes is also key in evaluating these patients. Urine analysis shows elevated sodium, potassium, chloride, calcium, and prostaglandin E2 excretion. Elevated levels of 24-hour urinary calcium can be used to rule out Gitelman syndrome (which is associated with low calcium excretion). In Bartter syndrome, serum/blood levels of renin and aldosterone are also elevated. An arterial blood gas in Bartter syndrome will show metabolic alkalosis. Confirmation of the diagnosis of Bartter syndrome can be made by genetic testing, such as next-generation sequencing, which includes testing for the following genes: SLC12A1, KCNJ1, CLCNKB, CLCNKA, BSND, and MAGED2 [4].

Based on the age of onset and severity, Bartter syndrome (BS) can be divided into two types: the classical variant, which manifests in early childhood and is less severe, and the antenatal variant, which occurs before birth and is frequently life-threatening. Bartter syndrome can also be divided into five types based on the genes involved, with Type I BS involving a mutation in the sodium chloride/potassium chloride cotransporter gene (NKCC2). Type II BS and Type III BS are caused by mutations in the renal outer medullary potassium channel (ROMK) and chloride channel-kb (CLC-Kb) genes, respectively. Type IV BS is caused by a loss of function mutation in the Bartter syndrome, infantile, with sensorineural deafness (BSND) gene. Mutations in the genes encoding chloride channel subunits (CLC-Ka and CLC-Kb) and the extracellular calcium ion sensing receptor can cause type V BS [5].

Other disorders like Gitelman syndrome, cystic fibrosis manifesting as a pseudo-Bartter syndrome, and Bartter-like syndrome have features overlapping with Bartter syndrome. Gitelman syndrome is also a rare, recessive, salt-wasting tubulopathy but with impaired salt reabsorption in the DCT (distal convoluted tubule). Previously, Bartter syndrome was distinguished from Gitelman syndrome by some clinical features, such as early onset, severity, presence of hypercalciuria, polyhydramnios, or growth retardation. Recent research has revealed that Bartter syndrome patients might experience delayed presentation, and some characteristics, including hypercalciuria, are not always present [6]. Cystic fibrosis and Bartter syndrome are rare hereditary diseases with overlapping clinical manifestations such as dehydration, failure to thrive, and similar electrolyte abnormalities [7]. Nephrotoxic agents (such as aminoglycosides, amphotericin B, and heavy metals) and anorexia nervosa have also been reported to be associated with Bartter-like syndrome [8].

The therapeutic approach for this rare syndrome varies among clinicians, who rely on personal experience, clinical presentation, and an understanding of the pathophysiology. There are few standard guidelines for treating Bartter syndrome, but they include potassium chloride replacement, a prostaglandin inhibitor (indomethacin), and a potassium-sparing diuretic (spironolactone, eplerenone, or amiloride). As potassium supplementation at higher doses is intolerable, a potassium-sparing diuretic is advised to keep the serum potassium in the physiologic range. Among the potassium-sparing diuretics, amiloride, which is a direct epithelial sodium channel (ENaC) inhibitor, is preferred over spironolactone and eplerenone, which work by blocking the aldosterone receptor. Aldosterone levels are usually low in these patients because they have hypokalemia. Due to this, some researchers have thought that amiloride might be better than aldosterone receptor blockers. Angiotensin-converting enzyme inhibitors (ACEI) can also treat hypokalemia in these patients, especially with coexisting proteinuria. However, caution should be taken to prevent possible hypotension and prerenal acute kidney injury (AKI) [8].

Some patients with Bartter syndrome will develop chronic kidney disease due to chronic hypokalemia, multiple episodes of dehydration, and nephrocalcinosis. However, the prognosis is usually good with strict adherence to the treatment and regular follow-up [9].

## Conclusions

Bartter's syndrome is a rare congenital salt-wasting renal tubular disease characterized by secondary hyperaldosteronism with hypokalemia and hypochloremic metabolic alkalosis. As with many other rare diseases, there are few clinical studies to guide diagnosis and therapeutic intervention in Bartter syndrome. Bartter syndrome usually presents in neonates, but this case report presents a rare case of adult-onset Bartter syndrome. Hence, Bartter syndrome must be considered a differential in cases with unexplained metabolic alkalosis. Diagnosis can be made on clinical grounds, serum electrolyte assessment, arterial blood gas analysis (ABG), and genetic sequencing. Administration of potassium chloride, a prostaglandin inhibitor, and a potassium-sparing diuretic are effective treatment modalities.
